# The dynamic evolution of the de Winter ECG pattern that is easily overlooked and life-threatening: a case report and literature review

**DOI:** 10.3389/fcvm.2025.1574829

**Published:** 2025-09-10

**Authors:** Yingmei Chen, Bao Chen, Lili Zhang, Wang Liao, Miao Wang

**Affiliations:** ^1^ ECG Room, Hainan General Hospital, Hainan Affiliated Hospital of Hainan Medical University, Haikou, China; ^2^ Department of Hospital Infection Management, Hainan General Hospital, Hainan Affiliated Hospital of Hainan Medical University, Haikou, China; ^3^ Department of Cardiology, Hainan General Hospital, Hainan Affiliated Hospital of Hainan Medical University, Haikou, China

**Keywords:** acute coronary syndrome, de Winter ECG pattern, coronary angiography, left anterior descending, upsloping ST-segment depression, electrocardiogram

## Abstract

**Background:**

Rapid diagnosis of patients with acute coronary syndrome (ACS) is crucial for saving their lives. The de Winter electrocardiogram (ECG) pattern is rare and is treated similarly to ST-segment elevation myocardial infarction (STEMI) and acute thrombotic occlusion of the coronary artery. The de Winter ECG pattern has been previously reported, but its dynamic evolution and characteristics have not been summarized.

**Methods:**

We reported two male patients who presented with de Winter ECG pattern at rest, and neither patient had a family history of hypertension, diabetes, or coronary heart disease. An urgent examination in our hospital showed elevated levels of cardiac troponin T. Both patients underwent emergency coronary angiography, which revealed subtotal proximal left anterior descending (LAD) stenosis. There was an improvement in chest tightness and pain after stent implantation. Serial ECGs before and after percutaneous coronary intervention showed dynamic evolution of ECG. A literature review was conducted to examine reported coronary angiography findings in patients with the de Winter pattern. The review focused on the dynamic evolution of the ECG and the accuracy of this pattern in diagnosing acute coronary artery occlusion (culprit vessel). It also highlighted the danger of the de Winter ECG pattern and the importance of emergency treatment.

**Results:**

Eighteen patients, including two of our patients, presented with the de Winter ECG pattern. Our two cases demonstrated two different forms of ST-segment dynamic evolution, with Case 2 being the only one among 18 cases that dynamically evolved into a life-threatening non-STEMI (NSTEMI). All cases were male patients with sudden chest pain. ECG examination showed an upward-sloping ST-segment depression with tall symmetrical T waves in the chest leads, and multiple follow-up ECGs revealed dynamic ST-segment evolution. Emergency coronary angiography showed occlusion of the LAD, left main artery (LMA), right coronary artery (RCA), first diagonal branch (D1), and left circumflex (LCX) artery as well as multiple vascular lesions. Most cases support subtotal stenosis or complete occlusion of the anterior descending artery. Timely identification of the de Winter ECG pattern and prompt transfer to the catheterization laboratory for emergency revascularization can be lifesaving and improve prognosis.

**Conclusion:**

These two cases and the literature review indicated that the de Winter ECG pattern is dynamically evolving. Its ECG pattern evolution is variable, progressing to STEMI, NSTEMI, Wellens, or even a normal. In patients presenting with chest pain, a de Winter ECG pattern, regardless of the subsequent dynamic evolution of the ECG, indicates the presence of severe coronary artery stenosis. The de Winter ECG pattern may be an early manifestation of ACS and requires urgent coronary angiography to save the patient's life and improve prognosis.

## Introduction

Acute coronary syndrome (ACS) is a clinical syndrome characterized by acute myocardial ischemia. It includes unstable angina, non-ST-segment elevation myocardial infarction (NSTEMI), and ST-segment elevation myocardial infarction (STEMI) (1). However, a portion of less commonly overlooked electrocardiograms (ECGs) do not conform to the typical electrocardiographic features of STEMI, nor do they differ from the common downward-sloping or horizontal ST-segment depression changes seen in NSTEMI. The main electrocardiographic features are upward-sloping ST-segment depression and symmetrical T-wave elevation, known as de Winter ECG patterns. This pattern is associated with acute thrombotic occlusion of the coronary artery and was first reported in 2008, specifically for proximal left anterior descending (LAD) artery occlusion (2).

In an era where coronary computed tomography angiography (CTA) and coronary angiography are the main diagnostic methods for myocardial infarction, traditional ECG still plays an irreplaceable role. The 12-lead ECG is the main diagnostic tool for detecting the de Winter patterns, especially in communities and primary medical institutions where coronary CTA and coronary angiography are unavailable. Early identification of the de Winter ECG patterns can be lifesaving by enabling rapid transfer of patients to the catheterization room for emergency revascularization to restore coronary blood flow. Various ECG patterns, especially atypical patterns and dynamic evolution of ECG, should be monitored frequently to ensure timely and accurate diagnosis.

Here, we present two patients with dynamic evolution of the de Winter ECG pattern, review the literature on its clinical characteristics, and emphasize the importance of repeated and continuous ECG monitoring in patients with acute typical chest pain to prevent treatment delays and adverse outcomes.

## Case presentation

### Case 1

**Figure 1:**
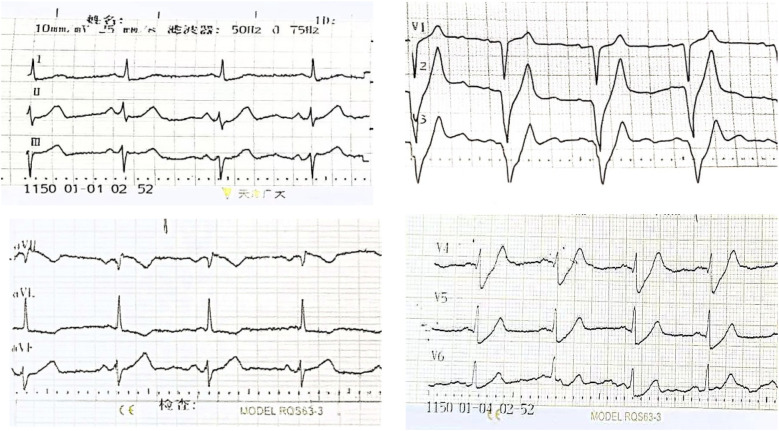
The initial ECG of Case 1 showed the de Winter ECG pattern: upsloping ST-segment depression of 0.1–0.4 mV in leads V2–V6 and persistent tall symmetrical T waves in leads V1–V4, along with an absence of R waves in leads V1–V3 and ST elevation in lead aVR.

A 61-year-old male with no history of hypertension, diabetes, or coronary heart disease was urgently referred to our hospital's emergency department. He initially presented to a local hospital with persistent chest pain for 4 h. The chest pain was described as a squeezing sensation in the chest area, accompanied by profuse sweating, palpitations, shortness of breath, radiating pain in the back and left upper limb, limb weakness, and dizziness, which persisted without relief. Physical examination showed body temperature of 36.2°C, pulse at 65 beats/min, respiratory rate at 20 breaths/min, and blood pressure at 104/70 mmHg. He had a clear consciousness and regular heart rhythm with no murmurs. Bilateral breath sounds were mildly coarse, accompanied by scattered moist rales in the lower lung fields. The abdominal examination revealed no abnormalities. The initial ECG examination in our hospital showed an upward-sloping ST-segment depression of 0.1–0.4 mV in leads V2–V6, tall symmetrical T waves in leads V1–V4, absence of R waves in leads V1–V3, and ST elevation in lead aVR (Figure 1).

**Figure 2:**
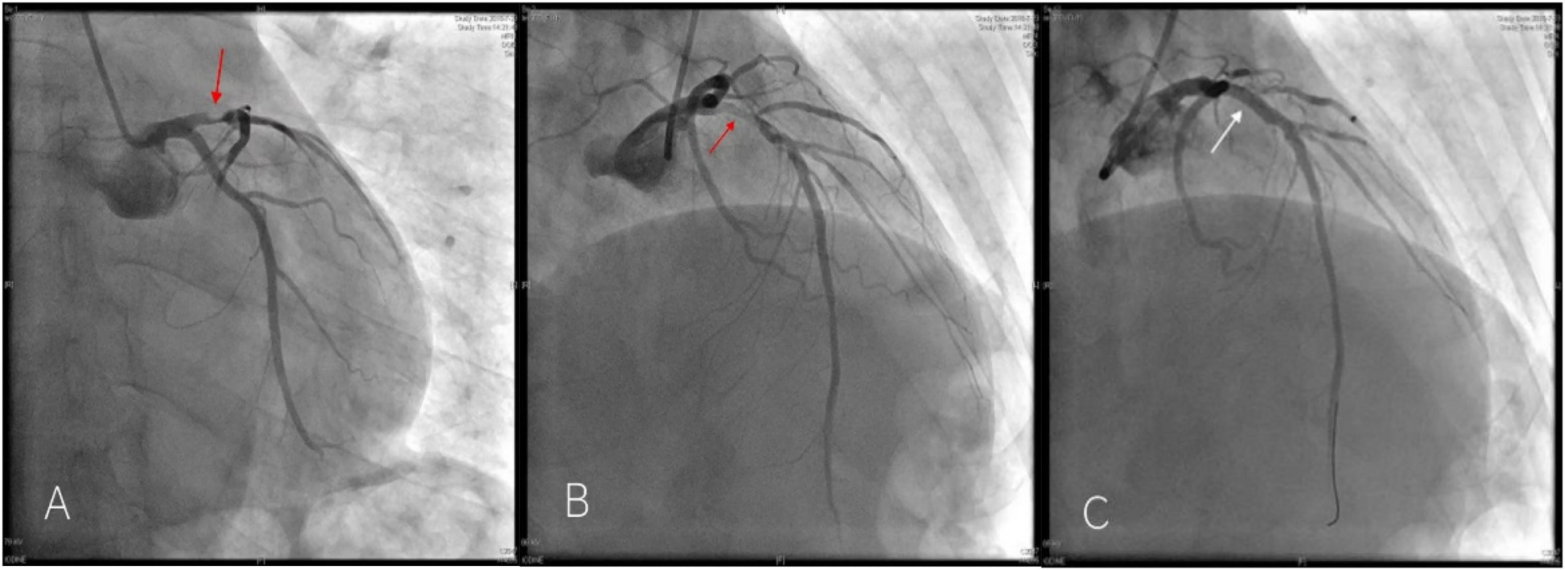
Emergency coronary angiography of Case 1 patient showed 75%–90% stenosis in the proximal LAD [**(A,B)** red arrow] and was successfully recanalized after stent implantation. [**(C)** white arrow].

**Figure 3:**
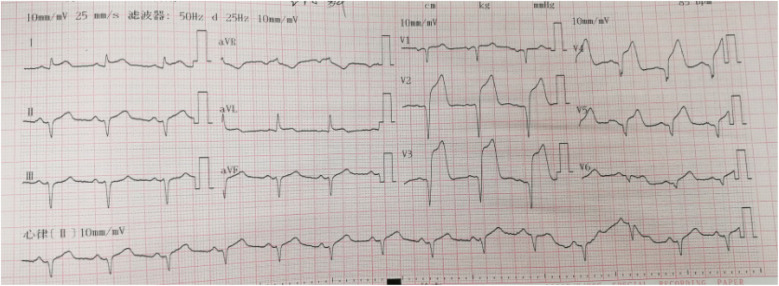
The second ECG of Case 1 showed an acute extensive anterior wall myocardial infarction: ST-segment elevation of 0.1–0.9 mV in leads II, III, aVL, and V2–V6. The most significant ST-segment elevation was observed in lead V3, accompanied by QS complexes in leads V1–V6, and upright T waves in leads V2–V6.

**Figure 4:**
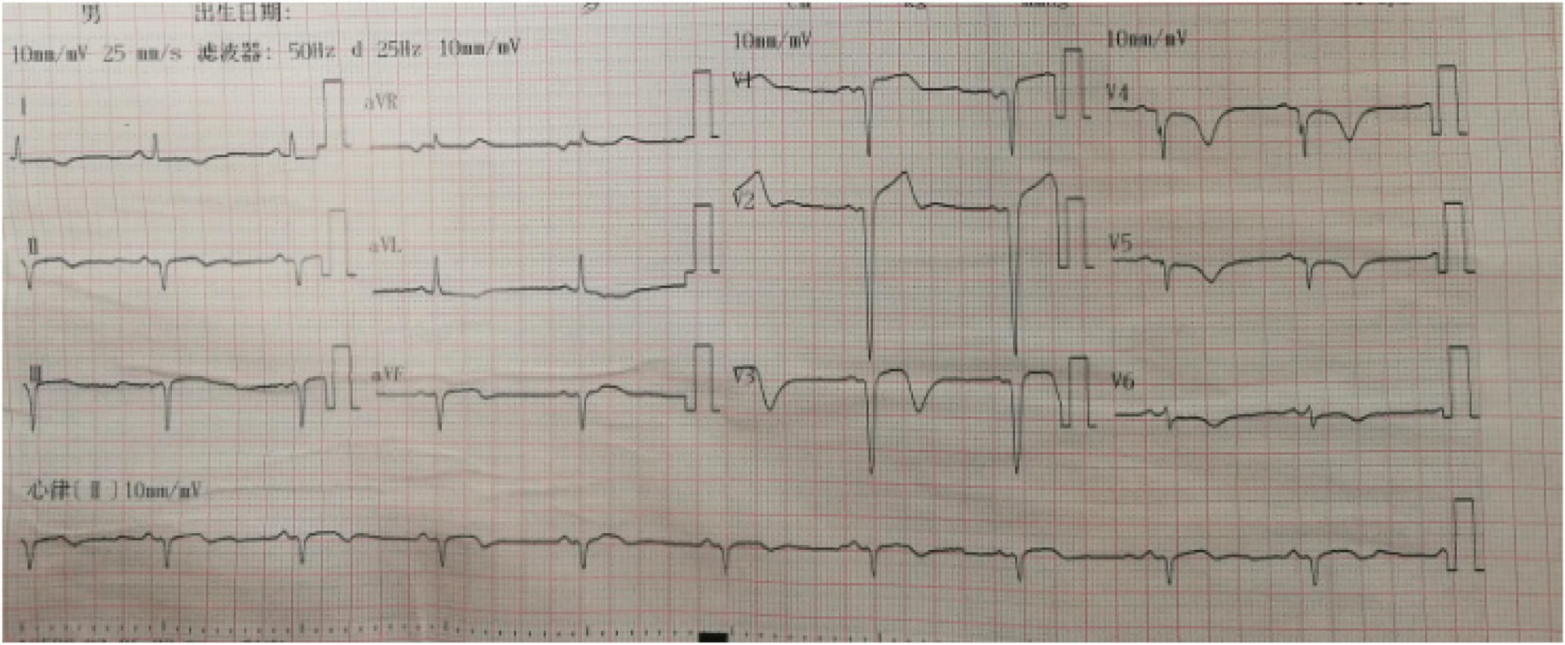
In the third ECG of Case 1, on the third day of PCI stent implantation, the ECG showed that the ST-segment gradually decreased to the isoelectric baseline, and pathological Q waves still existed in leads V1–V5, with inverted T waves.

Multiple rechecks of ECG showed a dynamic evolution of the ST-segment, which is gradually rising. In our hospital, emergent coronary angiography demonstrated 75%–90% stenosis in the proximal and middle segments of the LAD (Figures 2A,B). One stent was implanted, and the blood flow was restored to TIMI III (Figure 2C). Five hours after the initial ECG, QS complexes were observed on the follow-up ECG in leads V1–V6, with ST-segment elevation of 0.1–0.9 mV in leads II, III, aVL, and V2–V6, accompanied by upright T waves in leads V2–V6, consistent with a typical acute extensive anterior wall myocardial infarction (Figure 3). Ultrasensitive troponin T (TNT) was 10.0 µg/L, creatinine kinase (CK) was 5,382.4 U/L, CK-MB was 485.3 U/L, and D-dimer was 0.74 µg/mL. On the third day after percutaneous coronary intervention (PCI), the ECG showed that the ST-segment gradually decreased to the isoelectric baseline level, and pathological Q waves still existed in leads V1–V5, with inverted T waves (Figure 4). Echocardiography showed left heart enlargement, weakened motion of the anteroseptal segments, anterior wall, and apex, mild pulmonary hypertension, and a small amount of pericardial effusion. The patient had impaired left ventricular systolic and diastolic functions with left ventricular ejection fractions (LVEF) 35% and E′/A′ = 0.71. The patient received dual-antiplatelet therapy (aspirin 100 mg/day and ticagrelor 90 mg/12 h), and chest pain was relieved. The patient’s chest pain symptoms were significantly relieved, and he was discharged on the 14th day after PCI.

### Case 2

**Figure 5:**
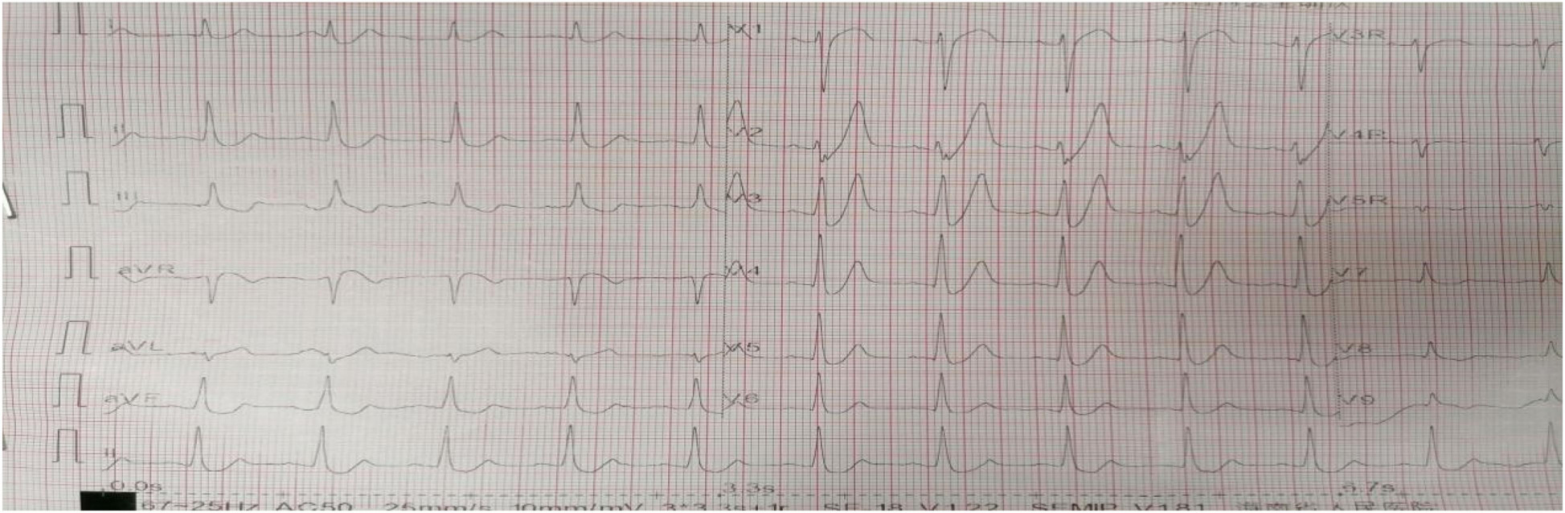
The initial ECG of Case 2 revealed the de Winter ECG pattern: upsloping ST-segment depression of 0.1–0.3 mV in leads V2–V6 and persistent tall symmetrical T waves in leads (V1–V4), ST-segment elevation of 0.1 mV in leads V1 and aVR.

**Figure 6:**
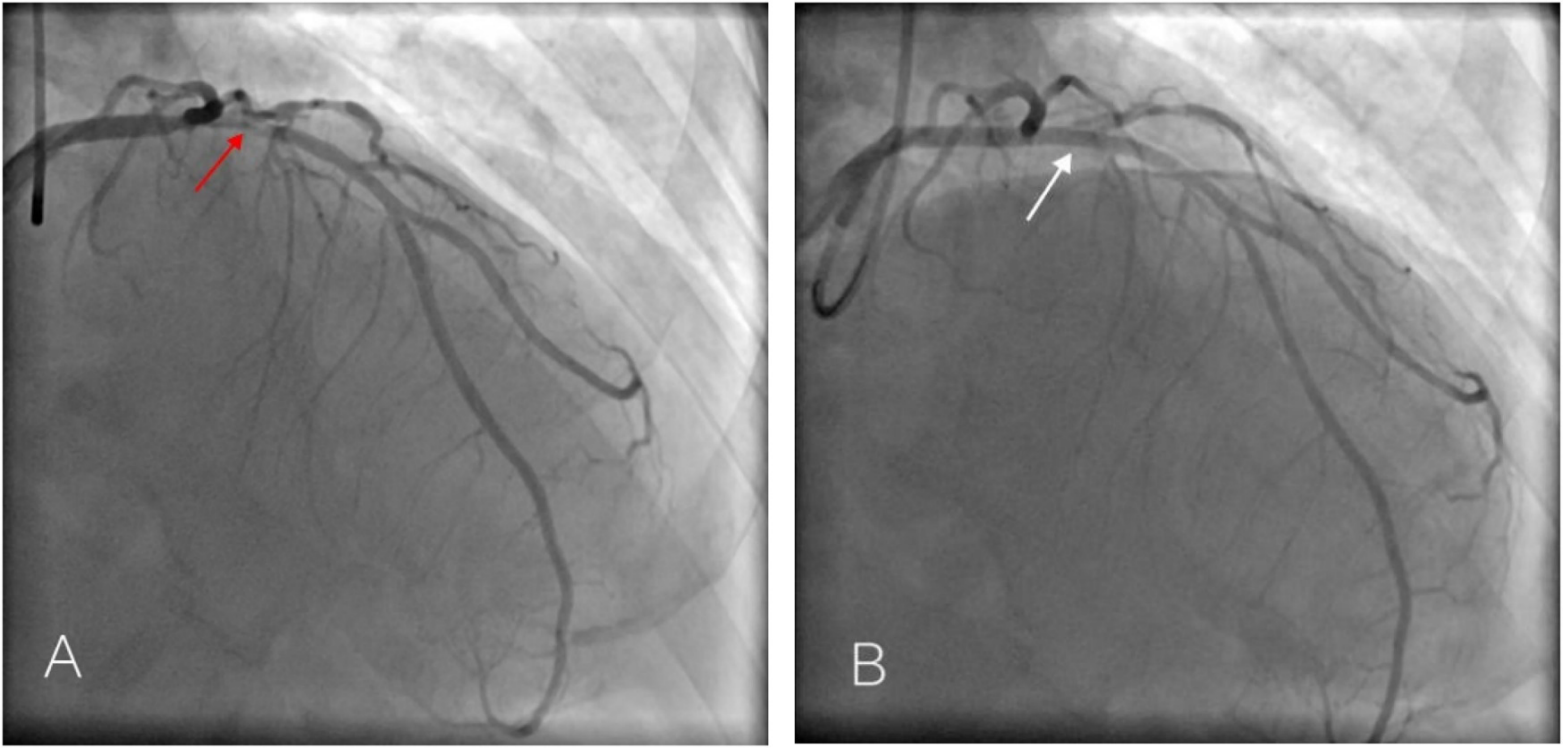
Emergency coronary angiography of Case 2 patient showed 85% stenosis in the proximal LAD [**(A)** red arrow] and was successfully recanalized after stent implantation [**(B)** white arrow].

**Figure 7:**
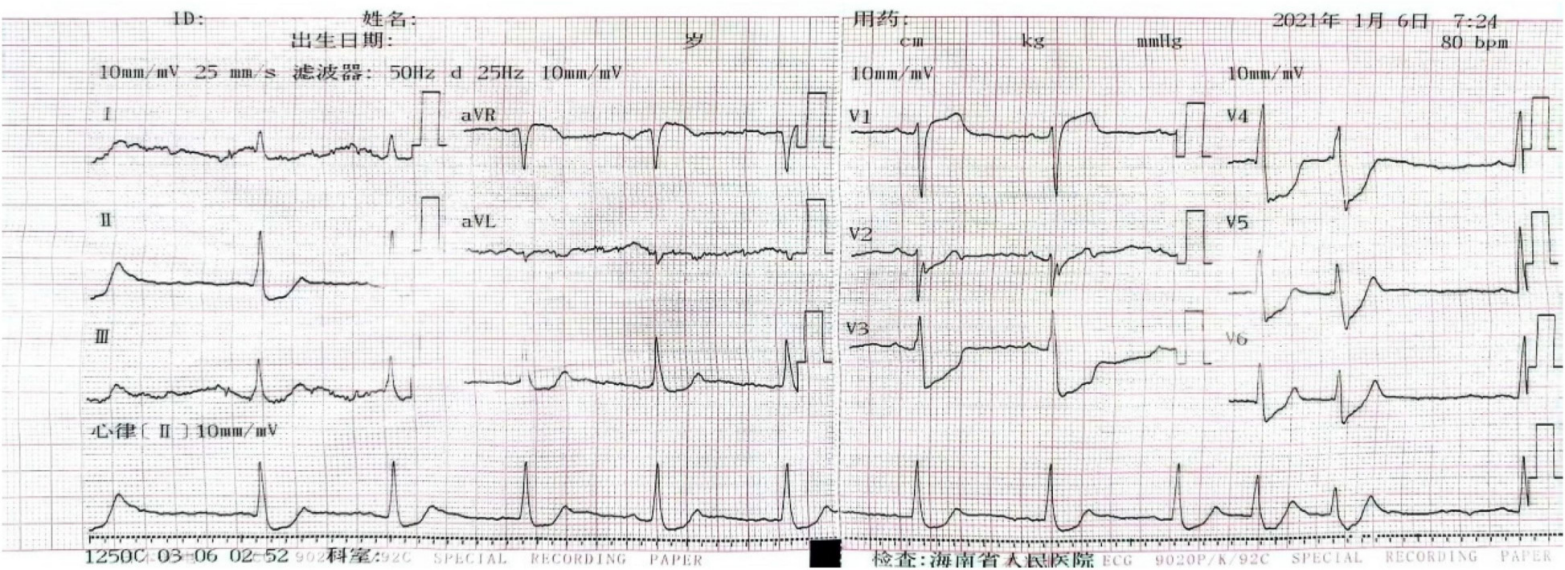
The second ECG of Case 2 revealed an NSTEMI different from de Winter ECG pattern: sinus rhythm; paired atrial premature beats; tall symmetrical T waves returning to normal; significant ST-segment horizontal and upsloping depression (0.1–0.7 mV) in leads II, III, aVF, and V2–V6; ST-segment depression in leads V3 and V4 which were most significant, and ST-segment elevation (0.1–0.2 mV) in leads aVR and V1.

**Figure 8:**
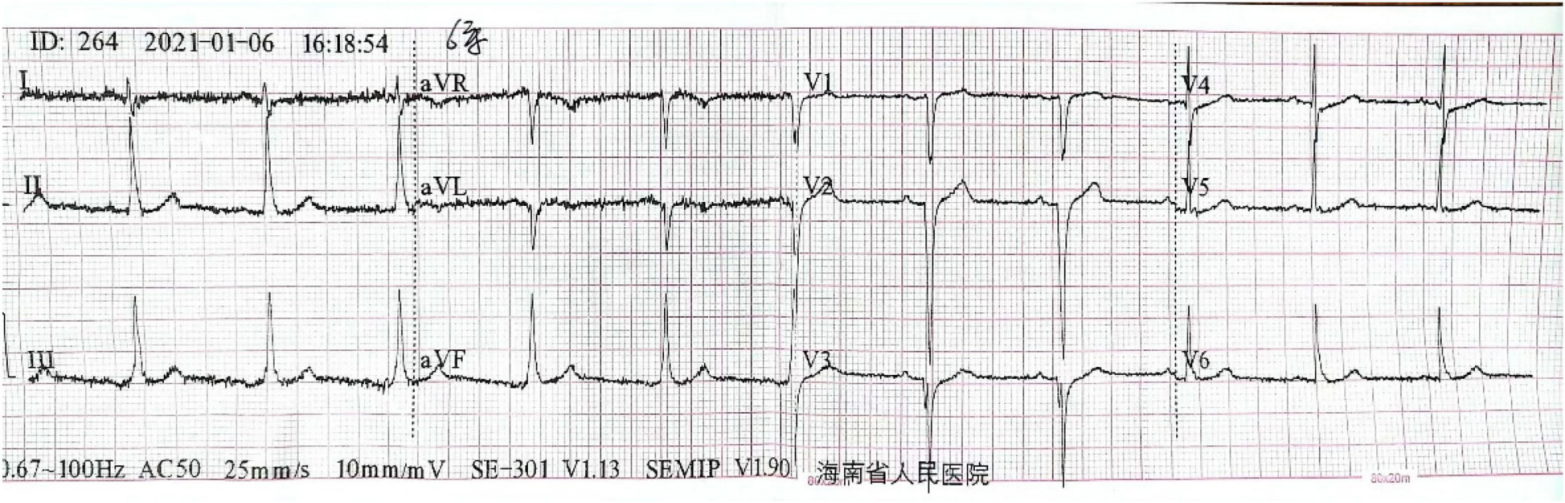
The third ECG of Case 2: V1–V3 leads were QS type, with ST-segment and T wave returning to normal.

A 55-year-old smoker without a history of hypertension, diabetes, or coronary heart disease presented to our emergency department with acute typical chest pain and continuous squeezing sensation behind the sternum, accompanied by profuse sweating and a feeling of impending death, which occurred during 2 h before waking. Physical examination showed body temperature of 37.0°C, pulse at 85 beats/min, respiratory rate at 20 breaths/min, and blood pressure at 130/80 mmHg. He had a regular rhythm and no pathological murmurs. Bilateral lung auscultation detected coarse breath sounds accompanied by mild wet rales in both lower lung fields. The results of the abdominal examination were unremarkable. The initial ECG showed 0.1–0.35 mV upward-sloping ST-segment depression with tall symmetrical T waves in leads V2–V6; 0.1–0.2 mV downward-sloping ST-segment depression in leads I, II, III, and aVF; and 0.1–0.15 mV ST-segment elevation in leads V1, and aVR. The ECG meets the diagnostic criteria of the de Winter ECG pattern (Figure 5). TNT was 6.13 µg/L, CK was 78.0 U/L, CK-MB was 27.6 U/L, and D-dimer was 0.91 µg/mL. Emergency coronary angiography indicates a stenosis of 85% in the proximal segment of the LAD, with a patent left circumflex (LCX) artery and right coronary artery (RCA) (Figure 6A). A 3.0 mm × 21 mm GuReater stent was implanted into the LAD (Figure 6B). The second ECG was taken 46 min later from the initial ECG, which showed a sinus rhythm; paired atrial premature contractions; a sharp T-wave regression; significant horizontal and upsloping ST-segment depression (0.1–0.7 mV) in leads II, III, AVF, and V2–V6; and ST-segment elevation in leads aVR and V1. ST-T morphological changes were different from the typical de Winter ECG pattern but similar to the “8 + 2” ECG pattern in acute left main artery (LMA) occlusion (Figure 7). After PCI, the patient received dual-antiplatelet therapy (aspirin 100 mg/day and ticagrelor 90 mg/12 h). Ten hours after the initial ECG, a third ECG showed QS type in leads V1–V3, with ST-segment and T wave returning to normal (Figure 8). Echocardiography showed left atrial enlargement, small pericardial effusion, mild mitral regurgitation, and normal left ventricular systolic function [ejection fractions (EF) = 68%]. The patient's chest pain improved, and the condition was stabilized. The patient's chest pain was significantly relieved, and he was discharged on the 5th day after PCI.

## Discussion

ECG is a crucial diagnostic technique that is rapid, cost-effective, accessible, radiation-free, and easy to track and observe. With the popularization, improvement, and development of electrocardiographic knowledge, ECG continues to provide valuable information for disease prevention, diagnosis, treatment, and prognosis in outpatient, emergency, and inpatient settings. In clinical diagnosis and treatment activities, it is difficult to detect the de Winter ECG pattern if the observation is limited to ST-segment elevation. The diagnostic criteria for the de Winter ECG pattern include the following: (1) upsloping ST-segment depression of 0.1–0.3 mV (1 mV = 10 mm) at the J-point below the isoelectric baseline in precordial leads (V1–V6), with tall, positive symmetrical T waves; (2) normal or slightly widened QRS wave morphology; (3) poor R-wave progression in the chest leads; (4) a narrow range of 0.1–0.2 mV ST-segment elevation in aVR. Among them, the V3 lead has the highest degree of upsloping depression in the ST-segment and the highest T-wave amplitude (2, 3). In this ECG pattern report, ST-segment depression accompanied by taller T waves is not limited to the chest leads (4) but may also occur in the inferior wall leads (5, 6). It is necessary to differentiate it from ST-segment depression caused by tachycardia and tall spike T waves caused by hyperkalemia.

The presence of ST-segment depression in a routine 12-lead ECG is commonly observed in non-ST-segment elevation myocardial ischemia, which is often associated with coronary atherosclerosis. It can also be seen in more critical conditions such as left main coronary artery occlusion. The de Winter ECG pattern can easily be confused with the ECG characteristics of typical LMA occlusion; typical ECG changes in LMA are ST-segment depression in multiple leads and ST-segment elevation in aVR. Upon close observation of our two typical cases and previously published reports regarding the de Winter ECG pattern, ST-segment depression was the most common finding, often manifesting as upsloping depression. Clinicians may mistakenly categorize all ECGs with ST-segment depression as stable myocardial ischemia, overlooking their significance, potentially leading to delayed treatment and more unfavorable outcomes. The typical de Winter ECG pattern primarily features upsloping ST-segment depression, suggesting a possibility of more severe myocardial ischemia compared with horizontal or downsloping ST-segment depression.

Previous studies have indicated that the de Winter ECG pattern is primarily observed in single-vessel coronary artery disease, which was useful for diagnosing an acute proximal LAD artery occlusion. This pattern remains static without significant evolution in typical acute ST-segment elevation myocardial infarction (STEMI) (7). In a recent retrospective study, the de Winter ECG pattern, also known as the de Winter syndrome, was identified as a transient and dynamic ischemic phenomenon in the progression of ACS. It reflects the formation of coronary artery thrombi and strongly suggests severe coronary artery disease. The culprit vessel is most commonly the LAD, although this ECG pattern has also been associated with other coronary artery occlusions (8). Previously, this mode was considered static, but in reality, it is transient and dynamic. The ECG pattern of STEMI and de Winter can evolve into one another. To further observe the dynamic evolution of the de Winter phenomenon, based on the summary of 2 typical cases collected in our hospital, 18 cases were selected that met the characteristics of the de Winter ECG pattern and showed dynamic evolution of the ECG. The basic literature and clinical characteristics of each case are shown in Table 1 (9–21). All 18 cases were male, with a median age of 55.0 years (*P*_25_ = 49.2, *P*_75_ = 65). Previous studies have observed that patients with de Winter ECG patterns were younger males with hypercholesterolemia (7, 22). Compared with patients with STEMI, the baseline characteristics of patients presenting with de Winter ECG patterns were found to be younger. Among the 18 cases, except for 1 case (10) where angiography was not performed to determine the location of vascular lesions, 10 cases were single-vessel occlusions, while 7 cases indicated multiple-vessel occlusions. Although there have been reports of patients with de Winter ECG pattern changes who underwent emergent coronary angiography showing no coronary stenosis and diagnosed with myocarditis subsequently confirmed by cardiac magnetic resonance imaging (23), de Winter ECG changes still indicate acute LAD occlusion to some extent. Such ECG patterns can suggest acute coronary artery occlusion, including the LMA, LCX, RCA, and first diagonal branch. There are also case reports related to multivessel disease.

**Table 1:** Summary of the clinical features of cases with dynamic evolution in the de Winter pattern.

Study	Gender	Age	Clinical presentation	Location of vascular occlusion	Evolution situation	Evolution time point
Cabezas et al. (9)	Male	53	Chest pain	D1	de Winter to anterolateral ischemia	After treatment
Pranata et al. (10)	Male	65	Chest pain	NA	de Winter to STEMI	Before treatment
Lin et al. (11)	Male	65	Chest pain, diaphoresis	LAD, LCX	de Winter to STEMI	Before treatment
	Male	50	Chest pain radiating to the left shoulder and back, accompanied by diaphoresis	LAD	STEMI to de Winter	Before treatment
Grandjean et al. (12)	Male	56	Chest pain	LAD	de Winter to STEMI	After treatment
Wang et al. (13)	Male	31	Chest pain	LAD	de Winter to STEMI	Before treatment
Lu et al. (14)	Male	51	Chest tightness and pain	LAD	de Winter to STEMI	After treatment
Zhang et al. (15)	Male	55	Chest pain	LAD, LCX	STEMI to de Winter	Before treatment
	Male	70	Chest pain	LAD, large diagonal branch	de Winter to STEMI	Before treatment
Chen et al. (16)	Male	47	Chest pain	RCA, LAD, LCX	STEMI to de Winter	After treatment
He et al. (17)	Male	76	Chest pain, along with dyspnea and sweating	LAD, RCA, LCX	de Winter to STEMI	Before treatment
Zhu et al. (18)	Male	in his 50s	Chest pain radiating to the left arm, sweating, and nausea	LAD	de Winter to Wellens	After treatment
Wang et al. (19)	Male	56	Chest pain	LAD	de Winter to STEMI to normal	Before treatment
Liu et al. (20)	Male	47	Chest pain	LAD, LCX, LMA	de Winter to STEMI	Before treatment
	Male	76	Chest pain	LMA	de Winter to STEMI	Before treatment
Huang et al. (21)	Male	34	Chest pain	LAD, LCX, RCA	STEMI to de Winter	Before treatment
This study	Male	61	Chest pain radiating to the shoulder and back and left upper limb, accompanied by diaphoresis	LAD	de Winter to STEMI	After treatment
	Male	55	Chest pain, diaphoresis	LAD	de Winter to NSTEMI	After treatment

D1, first diagonal branch; LAD, left anterior descending; LCX, left circumflex; RCA, right coronary artery; LMA, left main artery; NA, not available.

Currently, the pathophysiological mechanism and dynamic evolution of the de Winter ECG pattern remain elusive. The following mechanisms are speculated to be involved: (1) anatomical variations in Purkinje fibers leading to delayed endocardial conduction, or the lack of activation of sarcolemma ATP-sensitive potassium channels (2); (2) severe ischemia and hypoxia increasing the permeability of myocardial cell membranes, resulting in potassium ion efflux, delayed repolarization, and ischemic myocardial stunning; (3) an area of transmural ischemia so extensive that it does not generate injury currents toward the precordial leads, but only upwards toward the aVR lead; (4) the presence of collateral circulation or ischemic preconditioning, which protects the myocardium from transmural ischemic damage, thereby preventing ST-segment elevation; (5) subendocardial ischemia delaying endocardial repolarization, and prolonged epicardial repolarization leading to an increase in repolarization vectors in the same direction as a normal heart, forming a transmembrane action potential difference that causes ST-segment depression with tall symmetrical T waves; (6) the differential sensitivity of the epicardium and endocardium to ischemia, and the reaction of M-cells at the junction of the myocardial midlayer and endocardium to ischemia and hypoxia, which may be the main cause of the de Winter pattern (24); (7) ischemic mirror changes due to multivessel disease.

During subendocardial ischemia, prominent and elevated T waves can be recorded in the hyperacute phase. As the ischemia further progresses to transmural ischemia, the ECG evolves to show typical ST-segment elevation, sometimes even accompanied by pathological Q waves. He et al. (17) suggested that this dynamic evolution may be due to the gradual occlusion of coronary arteries, while Donato et al. (25) and Sunbul et al. (26) proposed that it could be related to changes in the anatomical structure of coronary arteries or the recruitment of collateral circulation. The distribution of ischemic myocardium and the progression of thrombosis may contribute to the occurrence of the de Winter ECG pattern. The evolution of de Winter ECG depends on factors such as unstable thrombosis, spontaneous thrombolysis, and coronary artery reperfusion. It reflects subtotal occlusion of the proximal or mid-LAD artery rather than total occlusion. If the thrombosis progresses to complete occlusion, it can evolve into STEMI (21).

Including our 2 cases, there were a total of 18 cases with dynamic ECG evolution. Among them, 10 cases evolved from the de Winter pattern to STEMI, as exemplified by our reported Case 1, where the ECG progressed from the de Winter pattern to the typical STEMI. Conversely, there were four cases where STEMI evolved into the de Winter pattern. Zhang et al. (15) reported two cases fitting these evolution patterns. In one case of acute extensive anterior wall myocardial infarction, the ECG evolved into a typical de Winter pattern just 8 min later. Emergency coronary angiography revealed a 90% stenosis in the proximal and mid-segments of the LAD and a 70% stenosis in the distal segment of the LCX artery. The other case showed the opposite trend, with the initially recorded de Winter ECG evolving into an acute anterior wall myocardial infarction after 1 h. Emergency coronary angiography indicated approximately 95% stenosis at the junction of the proximal and mid-segments of the LAD and 90% stenosis in the proximal and mid-segments of the large diagonal branch. In this study, there was one case of the de Winter pattern evolving into Wellens (18). This rare dynamic evolution might be associated with spontaneous recanalization after acute complete LAD occlusion. There was also one case where the de Winter ECG pattern evolved into STEMI and then into a normal ECG. This evolution could be due to incomplete occlusion caused by a coronary artery thrombus, with spontaneous thrombolysis of the occlusive thrombus leading to a transition from ST-segment elevation to an upsloping ST-segment depression characteristic of the de Winter ECG pattern and even normalization of the ECG (27). Dynamic evolution occurred in 11 cases before coronary angiography and in 7 cases after PCI with stent implantation. The dynamic changes in ECG may be related to timely blood reperfusion therapy. The intervals between dynamic evolutions varied, mostly occurring within minutes to hours, and even up to 3 days in one case (14). Yang et al. (28) suggested that delayed and atypical ECG changes in this pattern might be attributed to individual differences in coronary anatomy, recurrent ischemic attacks, and different stages of thrombus occlusion. Liu et al. (20) reported two cases of de Winter ECG pattern dynamically evolving into STEMI or even death in a short time. In one case, it evolved into an extensive anterior wall and high lateral wall myocardial infarction after 1.5 h. Emergency coronary angiography showed complete occlusion of the proximal LAD and LCX artery, with a blurred thrombus at the bifurcation of the LMA. Despite timely stent implantation, the patient died of refractory cardiac arrest 3 h later. In another case, ST-segment elevation gradually appeared after 25 min. Emergency coronary angiography indicated LMA occlusion, and the patient died of severe multiorgan failure despite stenting. They believed that in cases of acute complete LM occlusion, the de Winter ECG pattern can evolve into STEMI.

The electrocardiographic manifestations of NSTEMI in ACS are qualitative and risk-quantitative markers. The number and degree of ST-segment depression in leads are often related to the degree of ischemia and prognosis. Multi-lead ST-segment depression (greater than six-lead depression >0.1 mV), accompanied by aVR and/or V1 lead ST-segment elevation, often indicates the presence of severe coronary artery stenosis. Case 2 dynamically progressed from de Winter ECG pattern to NSTEMI. The dynamic evolution of ECG is mainly manifested as more significant ST-segment depression in multiple leads and ST-segment elevation in leads aVR and V1. Coronary angiography showed subtotal occlusion of the proximal segment of the LAD. Insufficient understanding of this condition could lead to underestimation of its risks and misclassification as stable angina pectoris, potentially missing the therapeutic window (29) and delaying diagnosis and treatment.

Not all patients with acute subtotal or total LAD occlusion exhibit typical STEMI ECG patterns. Approximately 2% of patients initially present with the de Winter ECG pattern (2). In cases of LAD occlusion, if spontaneous recanalization occurs, there may be a transition between STEMI and the de Winter ECG pattern. This could potentially progress to myocardial infarction before coronary blood flow is restored (7, 30, 31). If the thrombus undergoes spontaneous thrombolysis, the ECG may evolve back to normal. In Case 2, an elevated D-dimer level indicated spontaneous thrombolysis of the coronary thrombus. Pranata et al. (10) and Rao et al. (32) also suggested that the de Winter syndrome may be a thrombotic condition, supporting thrombolytic therapy. The ECG pattern of STEMI can shift to the de Winter ECG pattern due to spontaneous thrombolysis of coronary thrombi. Therefore, early intervention can prevent the progression from endocardial ischemia to transmural myocardial ischemia, thus avoiding the development of STEMI and irreversible myocardial damage. The importance of timely recognition of dynamic ECG changes lies in its ability to confirm acute coronary artery occlusion, guiding the direction of further patient management.

Electrocardiographers and emergency physicians must recognize the de Winter ECG pattern, not only because of its rarity but also due to the lack of typical ECG characteristics during its dynamic evolution, which often leads to clinicians' disregard. To our knowledge, patients presenting with the de Winter ECG pattern carry a high risk of death and adverse events. On 25 August 2018, the European Society of Cardiology (ESC) announced the globally unified definition, the Fourth Global Definition of Myocardial Infarction (FUDMI), at its annual meeting held in Munich, Germany, which first elaborated the de Winter ECG pattern and identified it as a very high-risk NSTEMI caused by anterior descending artery occlusion. The de Winter ECG pattern should be treated like other STEMI equivalents, with prompt revascularization therapy administered promptly. Therefore, early recognition of the de Winter ECG pattern is essential, and primary PCI with concurrent stent implantation is considered the optimal reperfusion strategy (33).

## Conclusions

We suggest the de Winter ECG pattern is dynamically evolving, transitioning between STEMI, Wellens, hyperacute T, NSTEMI, and a normal ECG. Although the 2023 ESC guidelines do not specifically mention the de Winter ECG pattern, they emphasize that patients with non-ST-segment elevation acute coronary syndrome (NSTE-ACS) who exhibit any very high-risk features—particularly dynamic ST-T changes—should undergo urgent coronary angiography and an early invasive approach as soon as possible (34). The de Winter ECG pattern should be regarded as an electrocardiographic equivalent of STEMI. It is important to keep in mind this rare and dangerous ECG pattern that needs attention. Emergency physicians, electrocardiogram physicians, and cardiologists should recognize these patterns and closely monitor dynamic ECG changes. These characteristics of ECG changes can aid in the rapid identification of patients, emphasizing the diagnostic and treatment philosophy that “time is cardiac muscle.” Urgent coronary angiography and prompt revascularization therapy should be performed. Early diagnosis of the de Winter ECG pattern can be lifesaving. Prompt identification of atypical ECG signs of ACS is essential for saving patients' lives and improving outcomes.

## Data Availability

The original contributions presented in the study are included in the article/Supplementary Material, further inquiries can be directed to the corresponding authors.
